# Adjusted Comparative Treatment Persistence of IL-23p19 Versus IL-17 Pathway Inhibitors in Adults With Plaque Psoriasis: A Systematic Review and Exploratory Meta-analysis of Real-World Time-to-Discontinuation Estimates

**DOI:** 10.7759/cureus.117280

**Published:** 2026-10-01

**Authors:** Deya S Migdady, Nooran Alshyyab, Hashem M Alkhalaileh, Zi Yong Goh, Yael Reingold, Omar Rababah, Tala M Zghayer, Yousef Alsalhi, Abdelrahman K Alsaidi, Mustafa G Alrabbai

**Affiliations:** 1 Internal Medicine, Ministry of Health, Irbid, JOR; 2 Faculty of Medicine, Jordan University of Science and Technology, Irbid, JOR; 3 Medicine and Surgery, University of Jordan, Amman, JOR; 4 School of Medicine, IMU University, Seremban, MYS; 5 Faculty of Medicine, International University of the Health Sciences, Basseterre, KNA; 6 Faculty of Medicine, Mutah University, Karak, JOR

**Keywords:** biologics, drug survival, il-17 inhibitors, il-23p19 inhibitors, psoriasis

## Abstract

Long-term biologic persistence is a clinically relevant real-world outcome in plaque psoriasis because it reflects sustained effectiveness, tolerability, acceptability, treatment burden, and access beyond short-term trial efficacy. We systematically reviewed adjusted real-world comparisons of treatment discontinuation between IL-23p19 inhibitors and IL-17 pathway inhibitors. We searched PubMed/MEDLINE, Scopus, Web of Science Core Collection, and Google Scholar from inception through August 8, 2026. Among 1,672 imported records, 580 duplicates were removed, 1,092 records were screened, 31 reports were sought, and 21 full texts were assessed. Six studies met the eligibility criteria; three provided adjusted time-to-event estimates for quantitative synthesis. Because only three observational studies contributed and one estimate was class-level whereas two were drug-level, the quantitative result is exploratory, and the certainty of evidence is very low. A random-effects restricted maximum likelihood (REML) model with Hartung-Knapp adjustment yielded a pooled standardized hazard ratio (HR) for discontinuation of 0.34 (95% CI 0.12-0.94; p = 0.045), with substantial heterogeneity (I² = 76.7%; τ² = 0.129) and a 95% prediction interval that crossed the null (approximately 0.05-2.16). The direction of effect favored IL-23p19 therapy across the three estimates, but magnitude varied. Drug-level sensitivity substitutions reduced estimated heterogeneity, while leave-one-out analyses showed marked small-sample instability. These findings are hypothesis-generating and do not establish comparative superiority.

## Introduction and background

Plaque psoriasis is a chronic immune-mediated inflammatory skin disease whose prevalence varies substantially by age and geographic region [1]. Moderate-to-severe disease can impair physical, emotional, and social functioning and usually requires long-term systemic therapy [2]. IL-23 promotes the expansion and maintenance of IL-17-producing cells, whereas IL-17 acts downstream on keratinocytes and other target cells; biologic therapies targeting these pathways achieve high rates of skin clearance [3].

IL-17 pathway inhibitors include secukinumab, ixekizumab, brodalumab, and bimekizumab, whereas IL-23p19 inhibitors include guselkumab, risankizumab, and tildrakizumab. These therapies differ in mechanism, dosing schedules, and other clinical characteristics, and randomized trials have demonstrated high short-term efficacy for agents targeting both pathways [4-6]. However, short-term efficacy does not fully capture treatment performance during routine long-term care.

Treatment persistence, or drug survival, is the time from treatment initiation until discontinuation. In clinical practice, it reflects multiple factors, including sustained disease control, adverse effects, adherence, treatment burden, patient and clinician preferences, and healthcare access [7-9]. Real-world studies have documented treatment discontinuation across biologic therapies, further supporting persistence as a clinically relevant long-term outcome [10,11]. Persistence therefore complements short-term efficacy outcomes and may help inform long-term treatment selection and sequencing.

Earlier reviews largely summarized drug-survival estimates across multiple biologic therapies [7,12,13]. The present review instead focused on adjusted direct comparisons between IL-23p19 and IL-17 pathway inhibitors, standardized the direction of hazard ratios (HR), and avoided treating correlated comparisons sharing the same reference cohort as independent observations. Bimekizumab was included in the eligible IL-17 pathway class definition, but no eligible study reported an adjusted direct bimekizumab-versus-IL-23p19 discontinuation estimate by the search date. Accordingly, this systematic review aimed to synthesize adjusted real-world comparative evidence for treatment discontinuation or persistence among adults with plaque psoriasis receiving IL-23p19 versus IL-17 pathway inhibitors, with quantitative synthesis restricted to sufficiently compatible adjusted time-to-event estimates.

## Review

Methods

Protocol and Reporting

This systematic review was conducted in accordance with the PRISMA 2020 guidelines [14]. The OSF project (https://osf.io/n9vrh) was created on August 9, 2026, after the August 8, 2026 search was completed, as a retrospective transparency archive containing the protocol, search strategies, screening materials, extraction workbook, analysis code, and figures [15]. It was not a prospective registration.

Eligibility Criteria

Eligible reports enrolled adult or predominantly adult cohorts with plaque or moderate-to-severe psoriasis and used a real-world observational cohort, registry, or claims design. They directly compared an IL-23p19 inhibitor (guselkumab, risankizumab, or tildrakizumab) with an IL-17 pathway inhibitor (secukinumab, ixekizumab, brodalumab, or bimekizumab) and reported an adjusted estimate of discontinuation, drug survival, persistence, or time to discontinuation. A small pediatric minority was permitted only when adult-specific estimates were unavailable and pediatric participants were negligible.

For quantitative synthesis, we first selected an adjusted time-to-event estimate for treatment initiation rather than switching. If a study reported several correlated eligible comparisons, we retained one estimate per independent cohort. Comparator selection was then applied: a reported class-level contrast was retained for the broad class question; otherwise, secukinumab was used as the common IL-17 comparator. Because this produced one class-level and two drug-level estimates, the resulting synthesis was treated as exploratory, and drug-level substitutions were examined in sensitivity analyses. Reviews, randomized efficacy-only trials, single-arm reports, psoriatic-arthritis-only or pediatric-only cohorts, safety-only or economic-only studies, conference abstracts without extractable data, and studies without an adjusted direct comparison were excluded. Non-HR or variance-incomplete estimates were retained only for narrative synthesis when otherwise relevant.

Information Sources and Search Strategy

PubMed/MEDLINE, Scopus, Web of Science Core Collection, and Google Scholar were searched from database inception through August 8, 2026; all searches were last run on August 8, 2026. OSF and PROSPERO were checked on the same date for existing or ongoing systematic review protocols and registration overlap.

The PubMed/MEDLINE search combined terms for psoriasis ("Psoriasis"[Mesh] OR psoriasis[tiab] OR "plaque psoriasis"[tiab] OR "moderate-to-severe psoriasis"[tiab]) with IL-23p19 terms ("Interleukin-23"[Mesh] OR "IL-23"[tiab] OR IL23[tiab] OR "IL-23p19"[tiab] OR IL23p19[tiab] OR guselkumab[tiab] OR risankizumab[tiab] OR tildrakizumab[tiab]) and IL-17 terms ("Interleukin-17"[Mesh] OR "IL-17"[tiab] OR IL17[tiab] OR secukinumab[tiab] OR ixekizumab[tiab] OR brodalumab[tiab] OR bimekizumab[tiab]) and outcome terms (persistence[tiab] OR "treatment persistence"[tiab] OR "drug survival"[tiab] OR "drug persistence"[tiab] OR discontinuation[tiab] OR "treatment discontinuation"[tiab] OR "time to discontinuation"[tiab] OR "treatment retention"[tiab] OR adherence[tiab] OR "real-world"[tiab] OR registry[tiab] OR cohort[tiab]), excluding animal-only studies.

The database-specific strategies were as follows: Scopus: TITLE-ABS-KEY(psoriasis OR "plaque psoriasis" OR "moderate-to-severe psoriasis") AND TITLE-ABS-KEY("IL-23" OR IL23 OR "IL-23p19" OR IL23p19 OR guselkumab OR risankizumab OR tildrakizumab) AND TITLE-ABS-KEY("IL-17" OR IL17 OR secukinumab OR ixekizumab OR brodalumab OR bimekizumab) AND TITLE-ABS-KEY(persistence OR "treatment persistence" OR "drug survival" OR "drug persistence" OR discontinuation OR "treatment discontinuation" OR "time to discontinuation" OR "treatment retention" OR adherence OR "real-world" OR registry OR cohort); Web of Science Core Collection: TS=(psoriasis OR "plaque psoriasis" OR "moderate-to-severe psoriasis") AND TS=("IL-23" OR IL23 OR "IL-23p19" OR IL23p19 OR guselkumab OR risankizumab OR tildrakizumab) AND TS=("IL-17" OR IL17 OR secukinumab OR ixekizumab OR brodalumab OR bimekizumab) AND TS=(persistence OR "treatment persistence" OR "drug survival" OR "drug persistence" OR discontinuation OR "treatment discontinuation" OR "time to discontinuation" OR "treatment retention" OR adherence OR "real-world" OR registry OR cohort); Google Scholar Search 1: "plaque psoriasis" (guselkumab OR risankizumab OR tildrakizumab) (secukinumab OR ixekizumab OR brodalumab OR bimekizumab) (persistence OR "drug survival" OR discontinuation); Google Scholar Search 2: psoriasis (guselkumab OR risankizumab OR tildrakizumab) (secukinumab OR ixekizumab OR brodalumab OR bimekizumab) ("adjusted hazard ratio" OR discontinuation OR persistence). No adjusted-HR terminology was imposed as a mandatory database-stage restriction; eligibility was determined during full-text review and extraction.

Study Selection

All records were imported into reference management software for deduplication and screening. Two reviewers independently screened titles and abstracts, then full texts, using predefined criteria. Disagreements were resolved by consensus. Full texts not retrieved due to closed access were documented as such rather than excluded for scientific reasons.

Data Extraction and Outcomes

Data extraction was performed using a standardized form capturing: study characteristics (author, year, country, design, data source), population (definition, sample size, biologic-naïve status), exposures (IL-23p19 and IL-17 drugs), outcomes (discontinuation, switching, persistence, definitions, follow-up), and effect estimates (adjusted HR, 95% CI, adjustment method). The primary outcome was adjusted treatment discontinuation, drug survival, persistence, or time-to-discontinuation. Secondary outcomes included switching, on-label persistence, and remission after discontinuation.

Risk of Bias

Risk of bias was assessed using the ROBINS-I tool for non-randomized studies [16]. Seven domains were evaluated: confounding, selection, classification of interventions, deviations from intended interventions, missing data, outcome measurement, and selective reporting. Overall risk was judged as low, moderate, serious, or critical. Risk-of-bias figures were generated using the robvis package [17].

Statistical Analysis

Only adjusted HRs with sufficient variance information were included in the quantitative synthesis. Effects were analyzed on the log-HR scale. Estimates reported in the reverse direction were inverted so that HR < 1 favored IL-23p19 inhibitors and HR > 1 favored IL-17 pathway inhibitors. Standard errors were calculated from 95% confidence intervals (CIs) as SE = [ln(upper CI) − ln(lower CI)]/(2 × 1.96).

To avoid treating correlated comparisons from the same cohort as independent observations, only one estimate per independent cohort was included in each meta-analysis. An adjusted time-to-event estimate from a treatment-initiation cohort was selected in preference to a switching cohort. When several eligible correlated comparisons were available within the same study, a reported class-level IL-23p19-versus-IL-17 contrast was retained for the broad class comparison; when no class-level estimate was available, secukinumab was selected as the common IL-17 comparator because it was available across the relevant drug-level studies. Consequently, the primary synthesis combined one class-level estimate with two drug-level estimates; this estimand mismatch was considered when interpreting the pooled result and was explored in sensitivity analyses.

The primary analysis used a random-effects model with restricted maximum likelihood (REML) estimation and Hartung-Knapp adjustment. For descriptive comparison and presentation of conventional inverse-variance weights, a fixed-effect model was also calculated. Heterogeneity was summarized using Cochran’s Q, I², and τ². Because only three independent estimates were available, a funnel plot was presented for descriptive purposes only; visual assessment of asymmetry was considered unreliable, no formal asymmetry test was performed, and no inference regarding publication bias or small-study effects was made from the plot [18]. Analyses were conducted in R version 4.6.1 (R Foundation for Statistical Computing, Vienna, Austria, https://www.R-project.org/) using the metafor package version 5.0-1 with rma.uni(yi, vi, method = "REML", test = "knha") [19]. A 95% prediction interval was obtained using predict() to describe the range of effects that might be expected in a comparable new setting. Sensitivity analyses examined alternative eligible comparator selections and leave-one-out models. Complete analysis code and session information are provided in the supplementary material.

Certainty of Evidence

The certainty of evidence was rated using the Grading of Recommendations Assessment, Development and Evaluation (GRADE) approach [20]. Evidence started as low due to observational designs and was downgraded for risk of bias, inconsistency, indirectness, or imprecision, and upgraded for large effects or dose-response gradients if applicable.

Results

Study Selection

The study-selection process is summarized in Figure 1. The searches identified 1,672 records: 344 from PubMed/MEDLINE, 858 from Scopus, 190 from Web of Science, and 280 from Google Scholar. After 580 duplicates were removed, 1,092 unique records underwent title/abstract screening. Of these, 1,061 were excluded and 31 reports were sought for retrieval. Twenty-one full texts were retrieved and assessed for eligibility, whereas 10 could not be retrieved. Following full-text eligibility assessment, six studies met the criteria for inclusion in the systematic review [21-26]. Three independent studies provided sufficiently compatible adjusted time-to-event estimates for the primary quantitative synthesis [21-23]. The 15 full-text reports excluded after eligibility assessment and their specific reasons for exclusion are presented in the Appendices.

**Figure 1:**
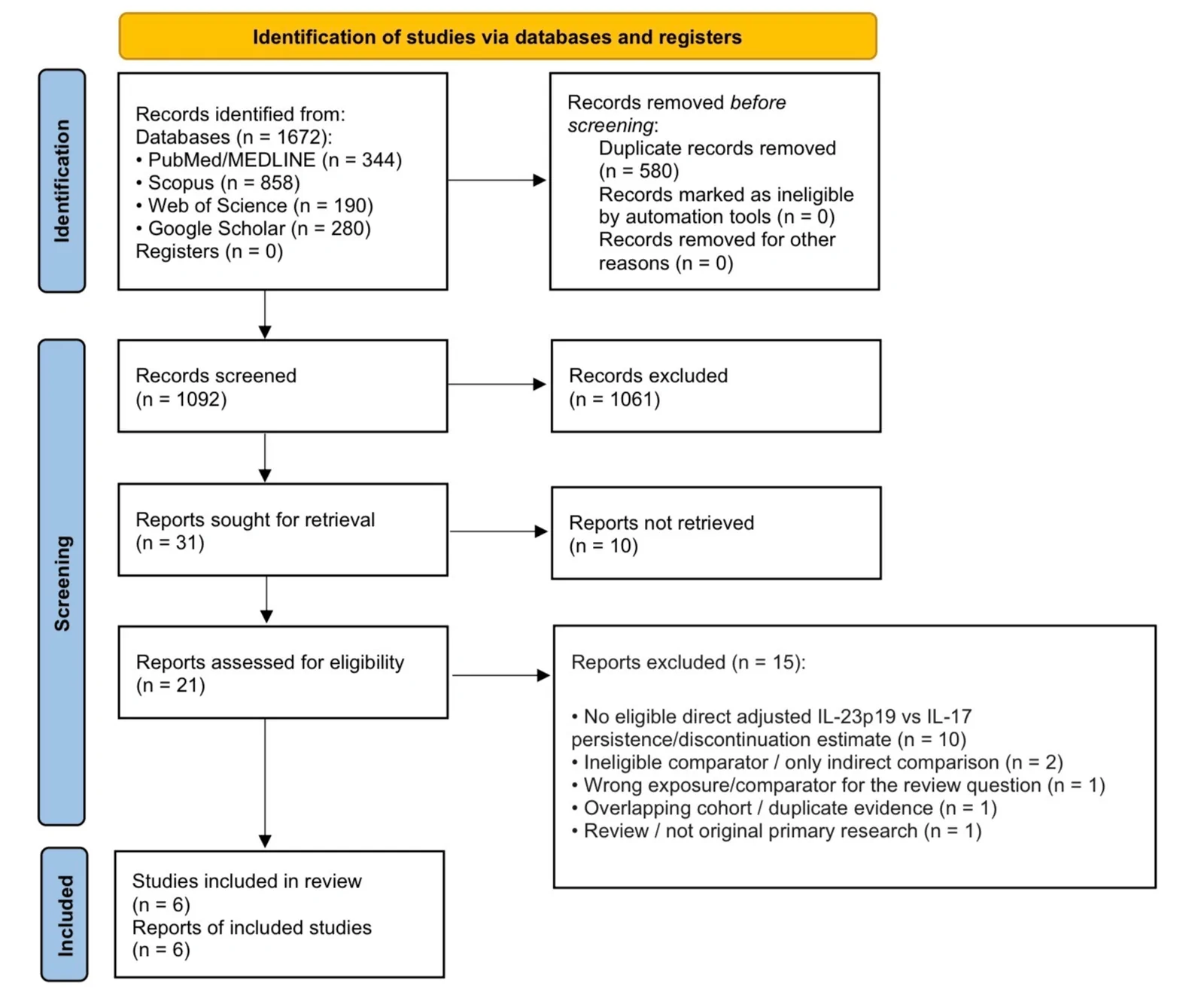
PRISMA flow diagram illustrating the study selection process PRISMA: Preferred Reporting Items for Systematic Reviews and Meta-Analyses.

Study Characteristics

Detailed clinical and methodological characteristics of the six included studies are presented in Table 1. The studies originated from Japan, Finland, Italy, the United States, and China. Data sources included administrative claims (Japan Medical Data Center, MarketScan), nationwide registers (Finland/Sweden), single-center clinical cohorts (Turin, Italy; Xiangya, China), and IQVIA PharMetrics Plus.

**Table 1:** Detailed characteristics of the six included studies Characteristics are presented according to the population and comparison relevant to this systematic review. Because some claims-based studies contained separate pairwise cohorts, sample sizes are reported by comparison rather than as a single unique study denominator. BRO: brodalumab, GUS: guselkumab, IL: interleukin, IXE: ixekizumab, JMDC: Japan Medical Data Center, KM: Kaplan-Meier, NR: not reported, PH: proportional hazards, PsO: psoriasis, RIS: risankizumab, SC: subcutaneous, SEC: secukinumab, TIL: tildrakizumab.

Study	Country	Data source	Design	Study period	Population	N	Mean age, years	Male, %	Biologic-naïve, %	IL-23p19 exposure	IL-17 comparator	Follow-up	Outcome definition	Adjustment/analysis method	Role in synthesis
Tada et al. [21]	Japan	JMDC claims	Retrospective cohort	2005-2022	Patients with PsO receiving IL inhibitors; 1.1% aged 0-17 years	1,481	48.1	70.0	Mixed	RIS	SEC, IXE, BRO	24 months	Discontinuation defined using a gap ≥150% of days supplied	Cox PH	Primary meta-analysis
Tskhvarashvili et al. [22]	Finland	Nationwide registers	Retrospective cohort	2018-2020	Bio-naïve adults with moderate-to-severe PsO	709	50.1	67.2	100	GUS	SEC, IXE	Up to 3 years	Time to treatment discontinuation, including switching	Covariate-adjusted Cox PH/survival analysis	Primary meta-analysis
Mastorino et al. [23]	Italy	Turin single-center cohort	Retrospective cohort	NR	Adults with moderate-to-severe PsO	1,057	54.7	65.2	63	IL-23 class: GUS/RIS/TIL	IL-17 class: SEC/IXE/BRO	24 months	Drug interruption/discontinuation	Multivariable Cox PH	Primary meta-analysis
Zhdanava et al. [24]	USA	MarketScan claims	Retrospective cohort	2016-2021	Adults with PsO initiating GUS, SEC, or IXE	GUS vs SEC: 3,516/6,066; GUS vs IXE: 3,805/4,674	47.6-47.7	51.7-51.9	54-59	GUS	SEC, IXE	18 months	Treatment persistence	Entropy balancing with weighted survival analysis	Narrative; different persistence estimand
Zhdanava et al. [26]	USA	IQVIA PharMetrics Plus	Retrospective cohort	2016-2023	Adults switching from another psoriasis-indicated advanced therapy	GUS: 935; SC IL-17i: 1,466	48.0	52-54	0; switcher population	GUS	Pooled SC IL-17i	24 months	On-label persistence from maintenance phase	Overlap propensity-score weighting; weighted KM and Cox models	Narrative; switcher population
Liu et al. [25]	China	Xiangya Hospital single-center cohort	Retrospective cohort	2019-2024	Patients with secondary failure to an IL-17A inhibitor	80	41.4	81.3	0	GUS, interclass switch	IXE/SEC, intraclass switch	28 weeks	Persistence/discontinuation after switching	Comparative descriptive analysis; predictor modeling	Narrative; secondary-failure switcher population

Reported cohort and treatment-group sizes varied substantially across the included studies and pairwise comparisons. Follow-up ranged from 28 weeks to up to 3 years. The Japanese study included 16 patients aged 0-17 years (1.1% of the cohort) [21]; the other included studies enrolled adult populations. Tskhvarashvili et al. used Finnish and Swedish register data, but the adjusted main-analysis estimate used in the present quantitative synthesis came from the Finland 2018-2020 cohort (n = 709) [22]. IL-23p19 exposures included risankizumab [21], guselkumab [22,24-26], and pooled IL-23 therapy [23]. IL-17 comparators included secukinumab and/or ixekizumab [21,22,24,25], brodalumab [21], pooled IL-17 therapy [23], and pooled subcutaneous IL-17 inhibitors [26]. Outcome definitions varied across studies and included claims-based treatment gaps, clinical treatment interruption or discontinuation, treatment persistence, and on-label persistence. No included study provided an adjusted direct bimekizumab-versus-IL-23p19 discontinuation estimate.

Adjusted Comparative Evidence

Adjusted and unadjusted comparative persistence estimates, their original direction, and eligibility for quantitative synthesis are summarized in Table 2.

**Table 2:** Comparative treatment-persistence and discontinuation outcomes and eligibility for quantitative synthesis CI: confidence interval, GUS: guselkumab, HR: hazard ratio, IL: interleukin, IXE: ixekizumab, RIS: risankizumab, SC: subcutaneous, SEC: secukinumab.

Study	Comparison	Outcome	Time horizon	Original effect measure	Adjustment	Effect estimate	95% CI	p-value	Original direction	Standardized interpretation	Primary pooling?	Reason if not pooled
Poolable adjusted discontinuation HRs												
Tada et al. [21]	RIS vs SEC	Discontinuation	Overall follow-up	HR	Adjusted	3.69	2.61-5.23	≤0.001	SEC vs RIS	Inverted HR ≈0.27; favors RIS	Yes	-
Tskhvarashvili et al. [22]	GUS vs SEC	Discontinuation	Up to 3 years	HR	Adjusted	3.81	2.42-6.01	<0.05	SEC vs GUS	Inverted HR ≈0.26; favors GUS	Yes	-
Mastorino et al. [23]	IL-23 class vs IL-17 class	Discontinuation	24 months	HR	Adjusted	0.54	0.37-0.78	0.001	IL-23 vs IL-17	Favors IL-23	Yes	-
Adjusted persistence estimates with a different estimand												
Zhdanava et al. [24]	GUS vs SEC	Persistence	18 months	HR	Adjusted	2.15	2.01-2.31	<0.001	GUS vs SEC	Favors GUS persistence	No	Persistence estimand differed from the prespecified discontinuation-HR synthesis
Zhdanava et al. [24]	GUS vs IXE	Persistence	18 months	HR	Adjusted	1.77	1.65–1.91	<0.001	GUS vs IXE	Favors GUS persistence	No	Persistence estimand differed from the prespecified discontinuation-HR synthesis

Across the three quantitatively compatible initiation cohorts, adjusted estimates consistently indicated a lower discontinuation hazard for IL-23p19 therapy, although effect magnitude varied. In the Japanese claims analysis, IL-17 agents showed higher adjusted discontinuation hazards than risankizumab: secukinumab HR 3.69 (95% CI, 2.61-5.23), ixekizumab HR 2.59 (95% CI, 1.80-3.75), and brodalumab HR 3.65 (95% CI, 2.47-5.38), all p ≤ 0.001. After standardization to the IL-23/IL-17 direction, these corresponded to HRs of 0.27, 0.39, and 0.27, respectively, favoring risankizumab [21].

In the Finnish bio-naïve registry analysis (2018-2020), discontinuation hazards were higher for secukinumab and ixekizumab than for guselkumab, with adjusted HRs of 3.81 (95% CI, 2.42-6.01) and 3.15 (95% CI, 1.84-5.37), respectively. Standardized IL-23/IL-17 HRs were 0.26 and 0.32, favoring guselkumab [22]. In the Italian single-center cohort of 1,057 patients, the adjusted class-level HR for IL-23 inhibitors versus IL-17 inhibitors was 0.54 (95% CI, 0.37-0.78; p = 0.001), representing a 46% lower hazard of discontinuation with the IL-23 class. Drug-level HRs versus secukinumab were risankizumab 0.42 (95% CI, 0.26-0.69; p = 0.001), guselkumab 0.49 (95% CI, 0.24-0.99; p = 0.046), and tildrakizumab 0.74 (95% CI, 0.33-1.65; p = 0.464) [23].

Evidence Not Included in Meta-analysis

Additional persistence and switcher evidence could not be combined with the primary analysis because the populations, estimands, or effect measures differed. The US MarketScan analysis provided complementary evidence on persistence rather than discontinuation. Guselkumab persistence was higher than secukinumab (HR 2.15; 95% CI, 2.01-2.31) and ixekizumab (HR 1.77; 95% CI, 1.65-1.91) at 18 months (both p < 0.001) [24].

The IQVIA PharMetrics Plus on-label persistence study reported that guselkumab was 78% more likely to maintain on-label persistence than pooled subcutaneous IL-17 inhibitors at 12 months, 87% more likely at 18 months, and 91% more likely at 24 months (all p < 0.001). Median on-label persistence was 32.8 months versus 14.2 months [26].

The Chinese single-center switcher study reported crude persistence rates in a special secondary IL-17A failure population: interclass switch to guselkumab discontinuation was 24.1% versus 2.7% for intraclass IL-17A switch [25]. The treatment-group comparison showed superior persistence with intraclass IL-17 switching, but this evidence was not quantitatively poolable with the primary initiation-cohort analysis because the population (secondary IL-17 failures), outcome metric, and effect measure differed substantially [25].

Secondary Outcomes

The Japanese claims study reported adjusted switching HRs versus risankizumab: ixekizumab 2.05 (95% CI, 1.26-3.33), secukinumab 2.67 (95% CI, 1.68-4.24), and brodalumab 2.77 (95% CI, 1.67-4.61), all p ≤ 0.01, indicating lower switching hazard with risankizumab [21]. The US MarketScan study reported remission rate ratios (exploratory proxy definition) favoring guselkumab versus secukinumab (RR 1.31) and ixekizumab (RR 1.40), both p < 0.001 [24].

Exploratory Primary Meta-analysis

The three independent adjusted estimates eligible for quantitative synthesis are shown in Figure 2. The exploratory synthesis combined two drug-level comparisons, risankizumab versus secukinumab from Tada et al. [21] and guselkumab versus secukinumab from Tskhvarashvili et al. [22], with one class-level comparison, pooled IL-23 versus pooled IL-17 from Mastorino et al. [23]. The fixed-effect estimate was HR 0.34 (95% CI, 0.28-0.43), while the primary random-effects REML model with Hartung-Knapp adjustment yielded HR 0.34 (95% CI, 0.12-0.94; p = 0.045). Heterogeneity was substantial (Q = 8.79, df = 2, p = 0.012; I² = 76.7%; τ² = 0.129). Fixed-effect study weights were 40.8%, 23.8%, and 35.4%, and random-effects weights were 35.1%, 30.8%, and 34.1% for Tada et al. [21], Tskhvarashvili et al. [22], and Mastorino et al. [23], respectively. The approximate 95% prediction interval was 0.05-2.16 and crossed 1. Given the small number of studies, substantial heterogeneity, and mixture of drug-level and class-level estimates, the pooled result should be interpreted as exploratory.

**Figure 2:**
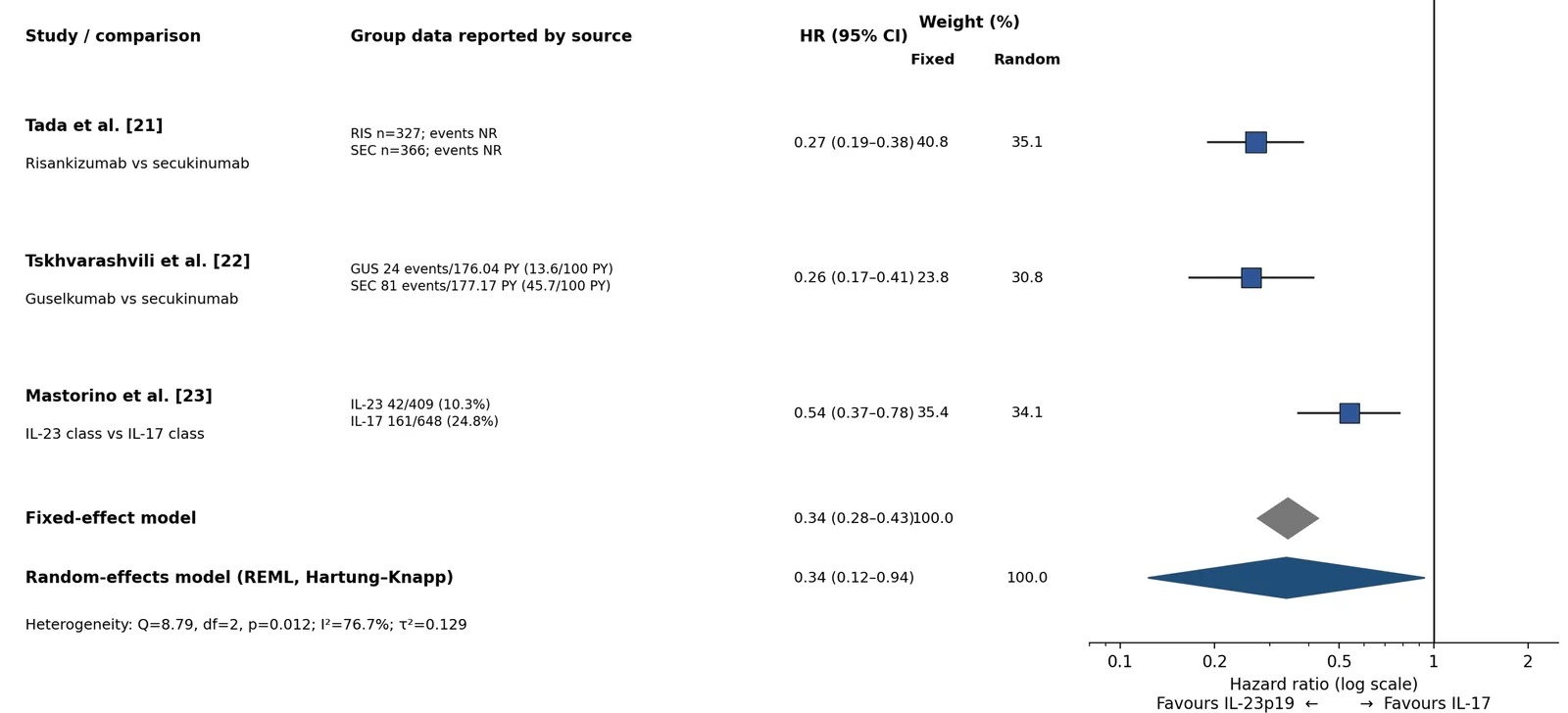
Forest plot of standardized adjusted treatment-discontinuation hazard ratios for IL-23p19 versus IL-17 pathway inhibitors Three independent adjusted estimates were included: risankizumab versus secukinumab from Tada et al. [21], guselkumab versus secukinumab from Tskhvarashvili et al. [22], and IL-23 versus IL-17 classes from Mastorino et al. [23]. Group-level data are shown as reported by the original studies. Fixed- and random-effects weights and pooled estimates are presented; the primary inference is based on the random-effects REML model with Hartung-Knapp adjustment. HR < 1 favors IL-23p19 therapy. CI: confidence interval, HR: hazard ratio, NR: not reported, PY: person-years, RIS: risankizumab, SEC: secukinumab, GUS: guselkumab, IL: interleukin.

All three standardized point estimates were below 1, corresponding to a lower discontinuation hazard with IL-23p19 therapy; however, the magnitude of association varied across studies. Because only three independent studies contributed and the prediction interval crossed the null, the pooled estimate should not be interpreted as a stable class-wide superiority estimate.

Sensitivity Analyses

The sensitivity analyses are summarized in Table 3. The direction of the pooled point estimate remained below 1 across alternative selections, but precision depended strongly on the chosen comparison. Replacing the Mastorino class-level estimate with its risankizumab-versus-secukinumab estimate produced HR 0.30 (95% CI 0.17-0.54; τ² = 0.005); replacing it with guselkumab versus secukinumab produced HR 0.29 (95% CI 0.16-0.54; τ² approximately 0). In contrast, the tildrakizumab-versus-secukinumab substitution was imprecise (HR 0.34, 95% CI 0.09-1.27; τ² = 0.161). Leave-one-out models retained point estimates below 1, but the two-study Hartung-Knapp intervals became extremely wide when either Tada or Tskhvarashvili was omitted. These analyses support directional consistency, not precision or model stability.

**Table 3:** Sensitivity analyses using alternative eligible comparisons and leave-one-out analyses All models used REML with Hartung-Knapp adjustment and one estimate per independent study. Alternative selections replaced only the indicated estimate; correlated estimates were never entered simultaneously. REML: restricted maximum likelihood.

Analysis	Pooled HR	95% CI	p-value	τ²
Primary: secukinumab comparator; class-level Mastorino estimate	0.34	0.12-0.94	0.045	0.129
Ixekizumab substituted in Tada only	0.39	0.16-0.93	0.043	0.081
Ixekizumab substituted in Tskhvarashvili only	0.36	0.14-0.91	0.042	0.103
Ixekizumab substituted in both drug-level studies	0.42	0.22-0.80	0.028	0.022
Brodalumab substituted in Tada	0.34	0.12-0.94	0.045	0.127
Risankizumab vs secukinumab substituted for Mastorino class estimate	0.30	0.17-0.54	0.013	0.005
Guselkumab vs secukinumab substituted for Mastorino class estimate	0.29	0.16-0.54	0.013	0.000
Tildrakizumab vs secukinumab substituted for Mastorino class estimate	0.34	0.09-1.27	0.072	0.161
Leave one out: Tada	0.38	0.004-37.19	0.228	0.215
Leave one out: Tskhvarashvili	0.38	0.005-30.43	0.219	0.204
Leave one out: Mastorino	0.27	0.22-0.33	0.007	0.000

Risk of Bias

Study-level ROBINS-I judgments are presented in Figure 3, while the distribution of judgments across ROBINS-I domains is summarized in Figure 4. All three studies contributing to the primary meta-analysis were judged at moderate risk of bias overall. Common concerns included residual confounding from unmeasured disease severity (PASI) in claims-based studies [21,24], confounding by indication and channeling to newer drugs, and differences in outcome definitions. The single-center study [23] had additional concerns regarding selection bias and observed-cases analysis. The Chinese switcher study [25] was judged at serious risk of bias due to the small sample size and highly selected population. None of the other studies were judged at serious or critical overall risk.

**Figure 3:**
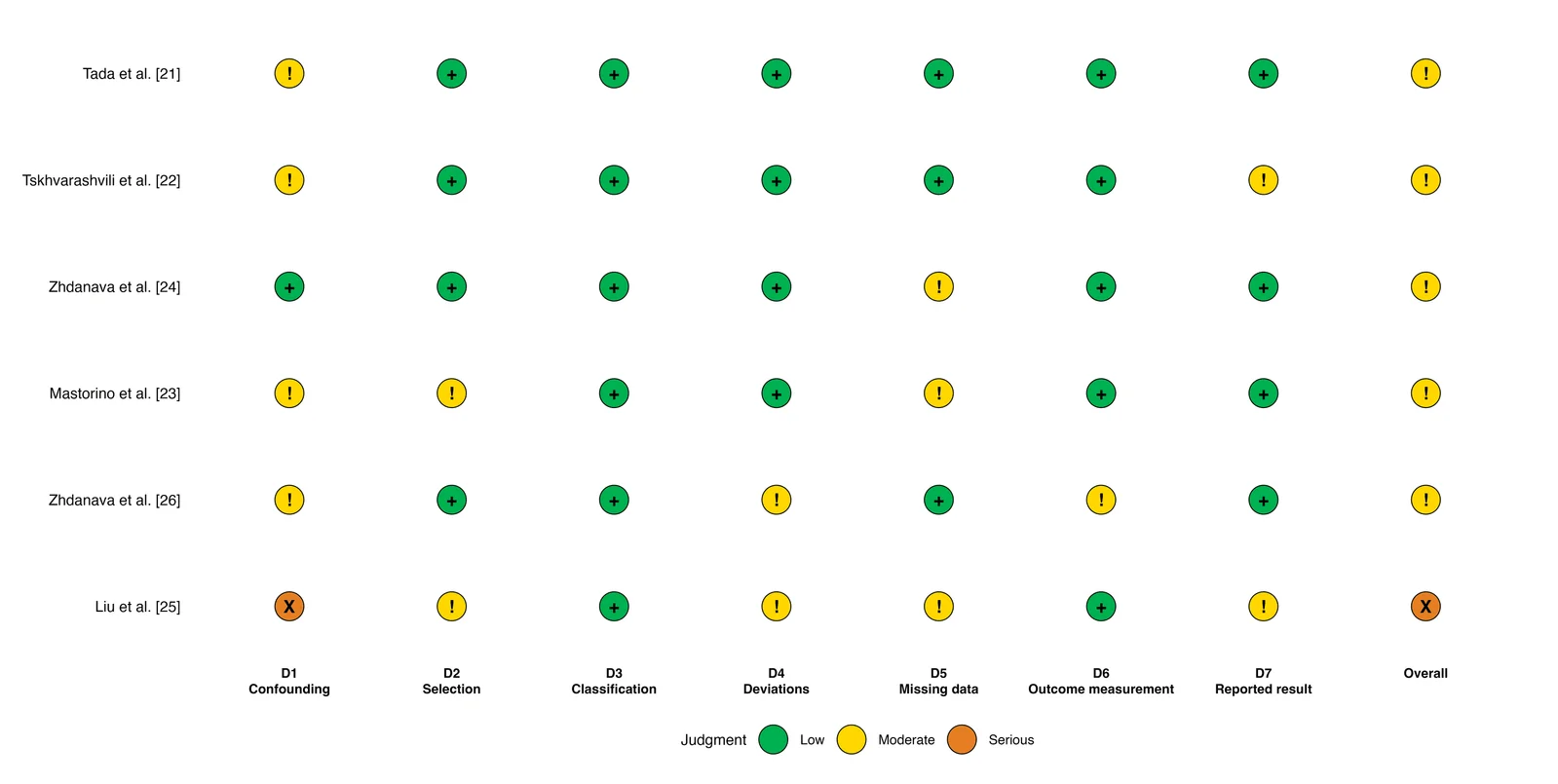
ROBINS-I traffic-light plot of study-level risk-of-bias judgments

**Figure 4:**
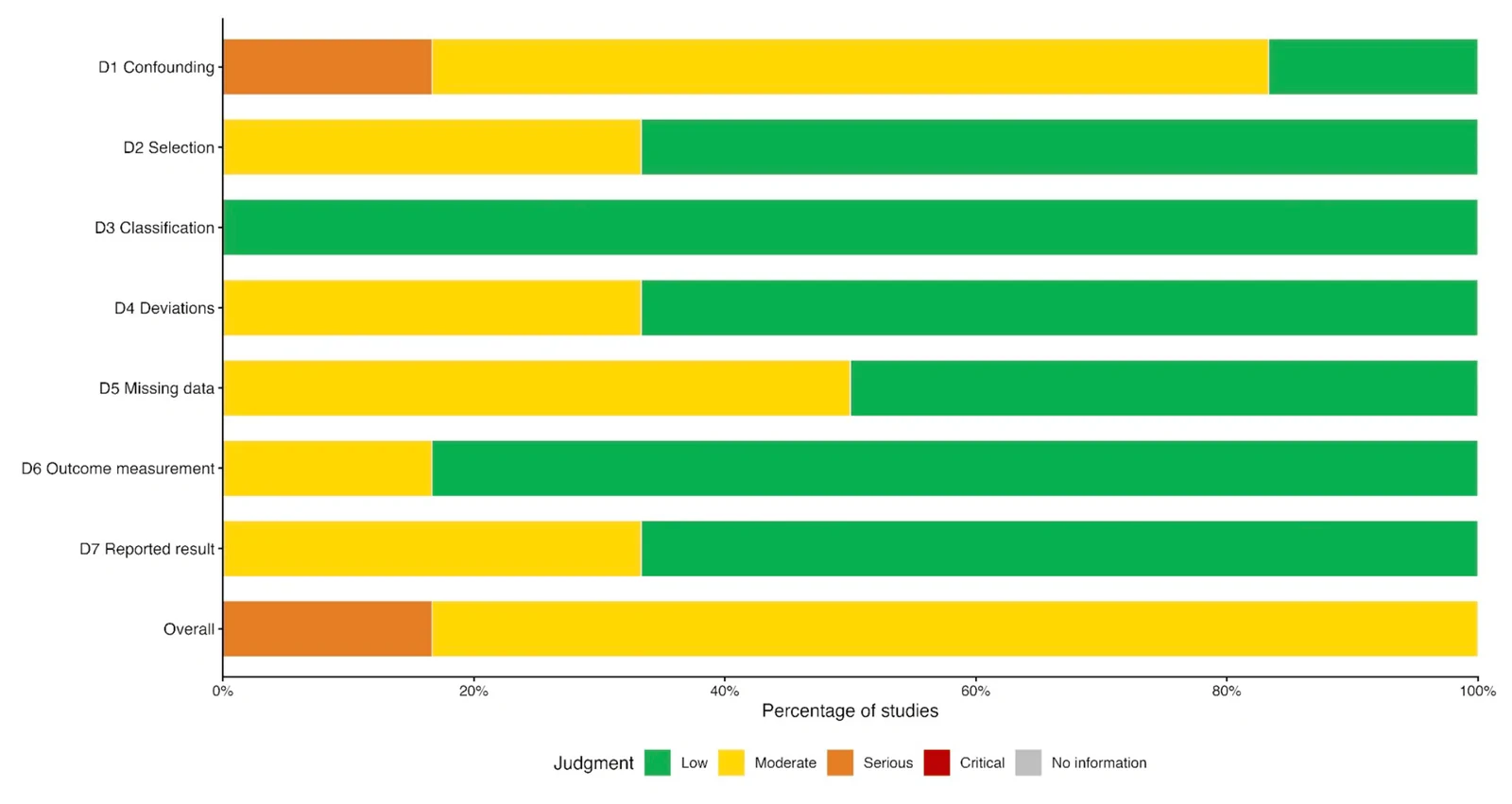
ROBINS-I summary plot across included studies

Only three independent studies contributed to the quantitative synthesis (k = 3). Given this very small evidence base, formal assessment of funnel-plot asymmetry was not considered appropriate, and no statistical test for small-study effects was performed [18]. In addition, the substantial between-study heterogeneity (I² = 76.7%) further limits the interpretability of any apparent asymmetry. The graphical assessment is presented in Figure 5; however, it should be interpreted descriptively only. Accordingly, the available data do not permit a reliable conclusion regarding the presence or absence of publication bias or other small-study effects.

**Figure 5:**
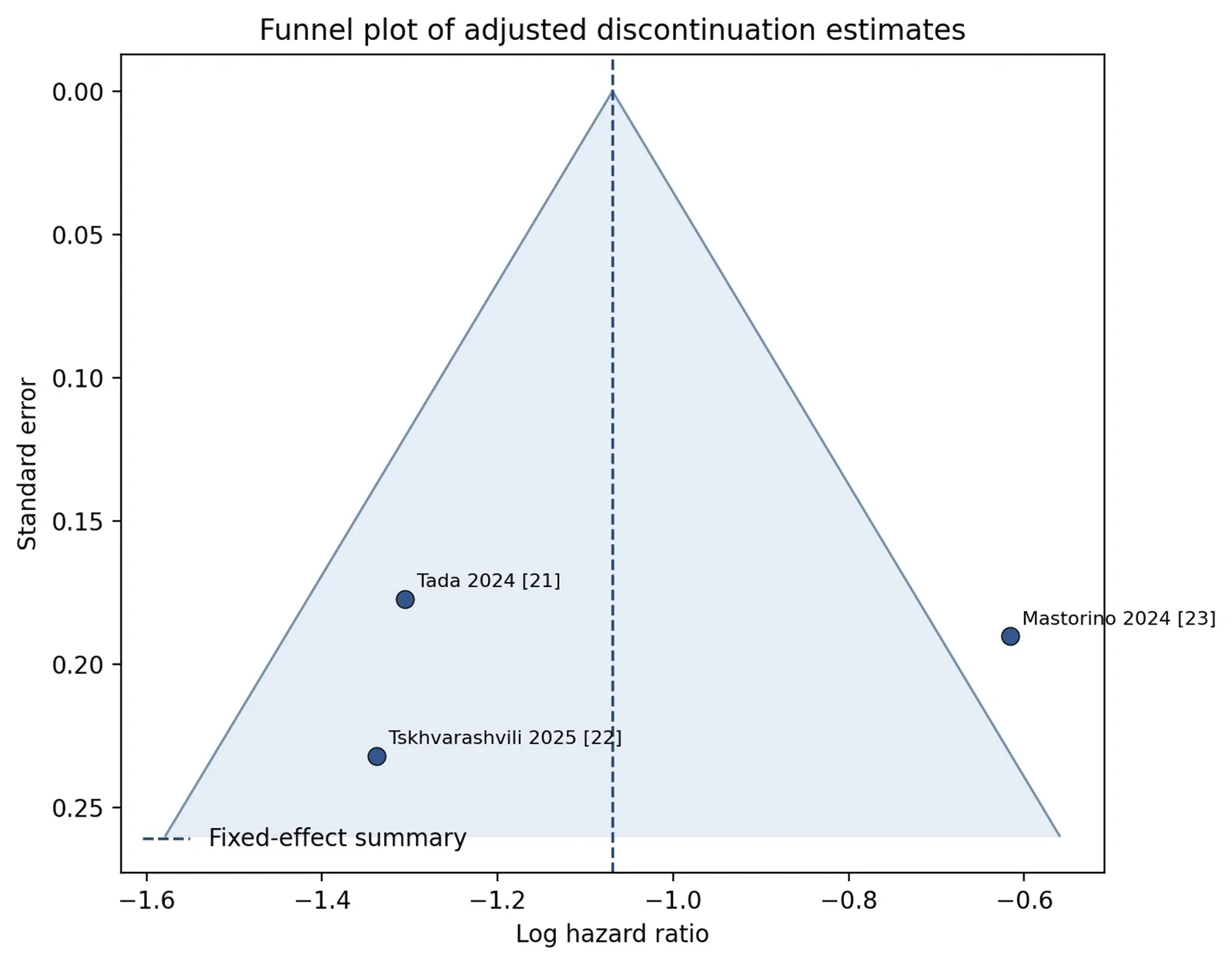
Funnel plot of adjusted treatment-discontinuation estimates Funnel plot of the three adjusted estimates from Tada et al. [21], Tskhvarashvili et al. [22], and Mastorino et al. [23]. The dashed vertical line represents the fixed-effect summary, and the shaded region represents pseudo-95% limits. Because only three studies were available, the plot is presented for descriptive visualization only and should not be interpreted as a reliable assessment of funnel-plot asymmetry.

Certainty of Evidence

The GRADE assessment for the primary outcome is summarized in Table 4. The certainty of evidence was rated very low. Evidence started low (observational designs) and was downgraded for risk of bias (residual confounding, unmeasured severity), inconsistency (substantial heterogeneity, I² = 76.7%), and imprecision (only three independent studies). The direction of effect was consistent, favoring IL-23p19 inhibitors, but certainty was insufficient for definitive conclusions.

**Table 4:** GRADE summary of findings for the primary quantitative outcome Observational evidence began at low certainty. ᵃDowngraded for residual confounding, confounding by indication, channeling to newer therapies, and incomplete measurement of disease severity. ᵇDowngraded for substantial between-study heterogeneity (I² = 76.7%). ᶜDowngraded for imprecision because only three independent studies contributed to the primary meta-analysis and the pooled confidence interval was wide. ᵈFormal assessment of publication bias or small-study effects was not performed because fewer than 10 independent studies were available. CI: confidence interval, GRADE: Grading of Recommendations Assessment, Development and Evaluation, HR: hazard ratio, IL: interleukin.

Outcome	Independent studies	Pooled effect	Heterogeneity	Risk of bias	Inconsistency	Indirectness	Imprecision	Publication bias	Certainty	Interpretation
Adjusted treatment discontinuation: IL-23p19 vs IL-17 pathway inhibitors	3	HR 0.34 (95% CI, 0.12-0.94)	I² = 76.7%; τ² = 0.129	Serious concernᵃ	Serious concernᵇ	Not serious	Serious concernᶜ	Not formally assessedᵈ	Very low	IL-23p19 inhibitors were associated with a lower hazard of discontinuation, but confidence in the effect is very limited

Discussion

This review found a consistent direction of effect across three adjusted initiation-cohort estimates, but effect magnitude varied and the evidence remains very uncertain. The pooled estimate combined two single-drug contrasts with one class-level contrast; this estimand mismatch appears to contribute materially to heterogeneity because τ² decreased substantially when the Mastorino class-level estimate was replaced with eligible drug-level comparisons. The 95% prediction interval crossed the null, and leave-one-out Hartung-Knapp analyses showed marked small-sample instability. The pooled estimate should therefore be regarded as exploratory rather than confirmatory evidence of class-wide comparative superiority.

This review extends earlier broad drug-survival syntheses [7,12,13] by restricting quantitative synthesis to adjusted comparative time-to-event estimates, standardizing effect direction, and avoiding the treatment of correlated comparisons from shared reference cohorts as independent observations. Differences among the included studies in population, previous biologic exposure, data source, follow-up, and outcome definition also limit comparability [21-26]. These methodological safeguards improve interpretation of the available evidence but cannot overcome the small and heterogeneous evidence base.

The evidence does not support an equally strong conclusion for every individual agent. Drug-level findings were driven mainly by risankizumab and guselkumab [21-23]. The only adjusted tildrakizumab-versus-secukinumab estimate was imprecise and not statistically significant (HR 0.74, 95% CI 0.33-1.65) [23]. Bimekizumab was included in the eligible IL-17 pathway class definition, but no eligible study reported an adjusted direct bimekizumab-versus-IL-23p19 discontinuation estimate by the search date. Additional persistence and switching studies provided clinically relevant context but involved different populations, estimands, or effect measures and were therefore not pooled with the primary discontinuation analysis [24-26]. Several factors may influence real-world persistence. Dosing frequency and treatment convenience may affect patient preferences and treatment burden [27,28], while differences in onset and durability of treatment response may also contribute [23,29]. Nevertheless, persistence is a composite outcome reflecting effectiveness, tolerability, adherence, patient and physician preferences, and healthcare access rather than efficacy alone [30]. Clinical interpretation should therefore also consider speed of response, comorbid psoriatic arthritis, safety, prior biologic exposure, dosing preferences, and access considerations [31,32].

Several potentially relevant studies provided descriptive or otherwise non-poolable evidence and therefore could not contribute to the quantitative synthesis [33,34]. Recent studies further broaden the evidence context but did not meet the direct adjusted-comparison requirement for quantitative synthesis. The 2025 BADBIR cohort evaluated age in relation to class-specific biologic survival rather than providing an eligible adjusted head-to-head IL-23p19-versus-IL-17 comparison [35]. A 2024 Italian multicenter cohort reported class- and drug-level survival without an extractable adjusted direct HR [36], while a 2026 Danish registry analysis reported class estimates against ustekinumab rather than a direct adjusted IL-23p19-versus-IL-17 contrast [37]. Their exclusion from the quantitative synthesis is documented in the Appendices.

Limitations include only three quantitatively compatible independent studies, observational confounding, inconsistent discontinuation definitions, mixed drug- and class-level estimands, substantial heterogeneity, an imprecise prediction interval, and small-sample Hartung-Knapp instability. Ten reports could not be retrieved. Future registry and claims studies should report standardized adjusted HRs with complete variance estimates, transparent handling of switching and discontinuation, and adequate adjustment for disease severity and prior treatment history. Head-to-head pragmatic trials or large registry-based emulated trials could provide more robust comparative evidence. Standardized definitions of discontinuation and persistence would facilitate meta-analysis.

## Conclusions

The available evidence is of very low certainty and supports an exploratory, rather than definitive, inference about comparative treatment persistence. Across three adjusted real-world initiation-cohort estimates, the direction of effect favored lower treatment discontinuation with IL-23p19 therapy, although effect magnitude varied; the pooled standardized HR was 0.34 (95% CI, 0.12-0.94), with substantial heterogeneity and a prediction interval that crossed the null. Importantly, this review provides a focused synthesis of adjusted direct comparisons, standardizes effect direction, and avoids double-counting correlated estimates from shared reference cohorts, thereby offering a clearer assessment of the available comparative persistence evidence than descriptive drug-survival estimates alone. However, the combination of two drug-level estimates with one class-level estimate and the small evidence base mean that the pooled result should be regarded as hypothesis-generating rather than evidence of class-wide superiority. Larger, consistently defined, and adequately adjusted head-to-head studies are needed to determine whether the observed persistence pattern is reproducible across individual agents and clinical settings.

## Appendices

Excluded full-text reports

**Table 5:** Full-text reports excluded after eligibility assessment and reasons for exclusion

Study	Reason for exclusion
Miyazaki (2024)	No adjusted direct IL-23p19-versus-IL-17 comparative HR
Ting (2024)	Descriptive drug survival; no extractable adjusted direct HR
Dapavo (2021)	Unadjusted narrative evidence and overlap with Mastorino 2024
Kishimoto (2023)	TNF-inhibitor index therapy; no eligible direct comparison
Alamer (2024)	Descriptive persistence; no adjusted direct HR
Xu (2024)	Explicitly unadjusted persistence estimates
Fitzgerald (2023)	Descriptive Kaplan-Meier evidence; no adjusted direct HR
Li (2023)	No adjusted direct comparative HR
Schmitt-Egenolf (2021)	IL-23p19 exposure sparse; no adjusted direct contrast
Armstrong (2023)	Switching descriptors without eligible adjusted persistence HR
Egeberg (2024)	Comparisons referenced to ustekinumab, not direct IL-23p19 versus IL-17
Gargiulo or Ibba (2024)	Descriptive multicenter survival; no adjusted direct HR
Kim (2026)	Class estimates versus ustekinumab; no reported direct adjusted contrast
Alabas (2025); BADBIR	Age contrasts within biologic classes, not a direct class comparison
Bucur (2021)	Narrative review; no primary comparative data
